# Diverse MicroRNAs‐mRNA networks regulate the priming phase of mouse liver regeneration and of direct hyperplasia

**DOI:** 10.1111/cpr.13199

**Published:** 2022-02-17

**Authors:** Rajesh Pal, Marta Anna Kowalik, Marina Serra, Cristina Migliore, Silvia Giordano, Amedeo Columbano, Andrea Perra

**Affiliations:** ^1^ 3111 Unit of Oncology and Molecular Pathology Department of Biomedical Sciences University of Cagliari Italy; ^2^ Department of Oncology University of Torino Torino Italy; ^3^ Candiolo Cancer Institute‐FPO, IRCCS Candiolo Italy

**Keywords:** Cyclin D1, hepatomitogens, MiRNAs, partial hepatectomy, transcriptomics

## Abstract

**Objectives:**

Adult hepatocytes are quiescent cells that can be induced to proliferate in response to a reduction in liver mass (liver regeneration) or by agents endowed with mitogenic potency (primary hyperplasia). The latter condition is characterized by a more rapid entry of hepatocytes into the cell cycle, but the mechanisms responsible for the accelerated entry into the S phase are unknown.

**Materials and methods:**

Next generation sequencing and Illumina microarray were used to profile microRNA and mRNA expression in CD‐1 mice livers 1, 3 and 6 h after 2/3 partial hepatectomy (PH) or a single dose of TCPOBOP, a ligand of the constitutive androstane receptor (CAR). Ingenuity pathway and DAVID analyses were performed to identify deregulated pathways. MultiMiR analysis was used to construct microRNA‐mRNA networks.

**Results:**

Following PH or TCPOBOP we identified 810 and 527 genes, and 102 and 10 miRNAs, respectively, differentially expressed. Only 20 genes and 8 microRNAs were shared by the two conditions. Many miRNAs targeting negative regulators of cell cycle were downregulated early after PH, concomitantly with increased expression of their target genes. On the contrary, negative regulators were not modified after TCPOBOP, but *Ccnd1* targeting miRNAs, such as miR‐106b‐5p, were downregulated.

**Conclusions:**

While miRNAs targeting negative regulators of the cell cycle are downregulated after PH, TCPOBOP caused downregulation of miRNAs targeting genes required for cell cycle entry. The enhanced *Ccnd1* expression may explain the more rapid entry into the S phase of mouse hepatocytes following TCPOBOP.

## Graphical abstract

A balance of pro‐ and anti‐proliferative signals is regulated by miRs in the priming phase of hepatocytes following pH‐induced liver regeneration, while miR deregulation leads only to pro‐proliferative signals in primary hyperplasia. This justifies the more rapid entry of hepatocytes into the cell cycle after TCPOBOP treatment.

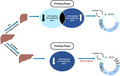

## 1 INTRODUCTION

Adult liver is normally quiescent and shows a very low level of hepatocyte division. However, most hepatocytes rapidly proliferate in response to a reduction in liver mass caused by physical, chemical, nutritional, vascular or virus‐induced liver injury.^1^ Although the mechanisms responsible for the exit from the quiescent state and the re‐entry into the cell cycle remain unknown, it has been proposed that the essential circuitry required for liver regeneration is encompassed by pathways activated by cytokines, growth factors and metabolic networks.^1^, ^2^, ^3^

More recently, several studies investigated the role of microRNAs (miRs) in liver regeneration following 2/3 partial hepatectomy (PH).^4^, ^5^, ^6^ Indeed, while miRs post‐transcriptionally regulate genes that orchestrate proliferation in development and cancer, their role in the proliferation of fully differentiated hepatocytes is still largely unknown. In this context, the finding that hepatocyte‐specific Dicer knockout transgenic mice developed normally, but exhibited enlarged livers compared to controls, strongly support the role for miRNAs in the control of hepatocyte proliferation.^7^ This study, together with the discovery that the association of miRNAs with different polysome fractions was altered during liver regeneration,^8^ raised the intriguing possibility that miRNAs might regulate the regeneration of this organ.

As shown by Shu et al.,^9^ upregulation of a cluster of miRNAs takes place between 0 and 4 h after PH, a time corresponding to the so‐called priming of hepatocytes,^10^, ^11^ characterized by refractoriness to DNA synthesis; conversely, downregulation of the vast majority of miRNAs associates with the transition G1‐S of the cell cycle and the recovery of liver mass. Accordingly, the expression of most cell cycle‐related genes is repressed for several hours after surgery. Similar findings were reported by Yin et al., who identified in rat liver transcription factors inhibiting the cell cycle, as early as 2 h after PH in rat liver.^12^ After the priming phase, hepatocyte DNA synthesis peaks at 24 or 36 h in rats and mice, respectively.^11^

Hepatocyte proliferation can be induced not only after cell death/loss but also following treatment with several xenobiotics or endogenous molecules (direct/primary mitogens), able to induce the entry of hepatocytes into the cell cycle in the absence of previous liver cell damage.^13^ Among the broad spectrum of chemical mitogens, it is remarkable that many of them are ligands of nuclear receptors of the steroid/thyroid superfamily, including 1,4‐bis[2‐(3,5‐dichloropyridyloxy)]benzene TCPOBOP (abbreviated thereafter as TCP). Studies with knockout mice have shown that the initial signalling elicited by liver regeneration and direct mitogens is different.^1^, ^2^, ^3^, ^14^, ^15^ Moreover, no change in the activation of transcriptional factors implicated in rat liver regeneration (such as, NF‐kB, AP‐1, STAT3) has been observed in nuclear receptor‐mediated hepatocyte proliferation.^13^ In this context, it should be also mentioned that the entry of hepatocytes into the S phase of the cell cycle in mice is robustly anticipated in mitogen‐treated animals (18 h instead of the 30–36 h required after 2/3 PH, with mitotic figures being evident at 24 vs. 48 h).^16^

Although existing studies analysed early responses of the mouse liver transcriptome at early times after treatment with TCP—an agonist of the constitutive androstane receptor (CAR)—^17^, ^18^ or soon after PH,^12^ the involvement of miRNAs as critical regulators in the priming phase of hepatocytes in these two proliferative conditions, has not been studied so far.

In an attempt to investigate whether deregulation of miRNA expression could play an essential role in the priming phase of hepatocytes and whether differences might exist between the two proliferative conditions (compensatory regeneration and direct hyperplasia), we performed a transcriptomic and miRNomic analysis on the liver of mice sacrificed 1, 3 and 6 h after 2/3 PH (liver regeneration) or after a single dose of TCP (direct hyperplasia).

## 2 MATERIALS AND METHODS

### 2.1 Animals and treatments

Guidelines for the Care and Use of Laboratory Animals were followed during the investigation. All animal procedures were approved by the Ethical Commission of the University of Cagliari and the Italian Ministry of Health. Three‐month‐old CD‐1 female mice (30 g) were fed a laboratory chow diet provided by Ditta Mucedola (Settimo Milanese, Italy) with free access to food and water. All experiments were performed in a temperature‐controlled room with alternating 12‐h dark‐light cycles. TCP (Sigma‐Aldrich), was dissolved in dimethyl sulphoxide/corn oil. A single dose of 3 mg/kg body weight was administered by gavage. PH was performed by removal of 70% of the liver mass as originally described by Higgins and Anderson.^19^ In the first set of experiments, mice were sacrificed 24, 36 and 48 h after PH or TCP. Bromodeoxyuridine (BrdU) (100 mg/kg) was injected intraperitoneally 2 h before sacrifice. To investigate the role of miRs in the priming of hepatocytes, mice were sacrificed 1, 3 and 6 h after each treatment. Three mice were used per group at each time point. Liver segments were fixed in formalin for histology or snap‐frozen in liquid nitrogen and kept at –80°C until use.

### 2.2 RNA and miR isolation

Total RNA was isolated with the RNeasy Plus Mini isolation kit (Qiagen) from 3 livers of untreated and treated mice, subjected to PH or TCP treatment. RNA was quantified by Nanodrop spectrophotometer (Thermo Scientific) and its integrity was evaluated by Agilent Bioanalyzer 2100. Only RNA samples with a RIN (RNA Integrity Number) ≥7 were included in the study.

### 2.3 Deep sequencing

For miR sequencing experiments, indexed libraries were prepared using 100 ng of total RNA as starting material, with a TruSeq Stranded Total RNA Sample Prep Kit and QIAseq miRNA Library Kit (Illumina Inc.), respectively. Libraries were sequenced (single‐end, 75 cycles) at a concentration of 8 pM/lane on the HiSeq 3000 platform (Illumina Inc.). Raw miRNA reads were preprocessed using FASTQC (Andrews, S. (2010) for quality control. Further, reads with Unique Molecular Identifiers (UMI) and low‐quality base calls were trimmed off using UMI‐tools^20^ and Trim Galore,^21^ respectively. Processed reads were mapped to the reference mouse genome build (mm10) downloaded from UCSC (https://genome.ucsc.edu/index.html) using Bowtie.^22^ The R/Bioconductor package “DESeq2” was used to identify differentially expressed genes and miRs. Data were filtered according to read count value (threshold ≥6 reads). Only miRs having an adjusted P‐value of ≤0.05) and fold change value of 1.3 were considered for further analysis.

### 2.4 Microarray

For time‐course expression profiling, total liver RNA was extracted and purified from the liver of three animals before (t=0) or 1, 3 or 6 h after treatment. For the gene expression profile, RNA was amplified (TotalPrep RNA Amplification Kit; Illumina Inc., San Diego, CA), labelled and hybridized on Illumina microarray Mouse WG‐6 v2.0 Gene Expression BeadChip (Illumina Inc., San Diego, CA, USA), including 45,281 specific oligonucleotide probes.

The intensity files were loaded into the Illumina BeadStudio software version 3.0.19.0 (Illumina Inc.) and BRB Array Tools version 4.6.0 for quality control and gene expression analysis. First, the quantile normalization algorithm was applied to the data set. Only genes whose expression differed by at least 1.5‐fold from the median in at least 20% of the arrays and characterized by the 50th percentile of intensities >300 were retained. The false discovery rate–adjusted P‐values were calculated using the Benjamini‐Hochberg procedure. To identify the differentially expressed genes, F‐test and Multivariate Permutation Test were applied. Further, the genes were filtered based on their fold change values (±1.5).

### 2.5 miR Target gene Prediction

The R package multiMiR^23^ was used to predict and validate miR‐mRNA target interactions. List of DE genes and miRs passing the cut‐off value was used as input. Among the databases in the multiMiR package, the validated databases (miRecords, miRTarBase and TarBase) and top 10% results of the predicted database (DIANA‐microT ElMMo, Microcosm, miRanda, miRDB, Pictar, PITA, TargetScan) were used for analysis.

### 2.6 qRT‐PCR analysis

The same cDNA used for gene sequencing was used also for qRT‐PCR analysis. Total RNA was retro‐transcribed using the High Capacity cDNA Reverse Transcription Kit (Thermo Fisher Scientific). Gene expression analysis of *Ccnd1*, *Cdkn1a*, *Cyp2b10*, *Socs3*, *Gadd45a* and *Gadd45b* was performed using specific TaqMan probes (Thermo Fisher Scientific, 4369016). Each sample was run in triplicate and all measurements were normalized to *β*‐*actin*. Relative *mRNA* expression analysis for each gene was calculated by using the 2^−ΔΔCt^ method.

Analysis of miRNA expression. cDNA was synthesized using the TaqMan^®^ MicroRNA ReverseTranscription Kit (4366596). qRT‐PCR amplification was performed with the reverse transcription product, TaqMan^®^ 2X Universal PCR Master Mix, No AmpErase ^®^UNG (4324018) and miR specific primers. The endogenous control *sno202* was used to normalize miRNA expression levels.

### 2.7 Histology and Immunohistochemistry

Liver sections were fixed in 10% of buffered formalin and processed for immunohistochemistry (IHC). For BrdU detection, paraffin‐embedded 4 µm sections were deparaffinized, treated with HCl 2N for 1 h and then with 0.1% trypsin at 37°C. Sections were sequentially incubated with goat serum (Abcam), mouse monoclonal anti‐BrdU antibody (Becton Dickinson) and with Dako EnVision+^®^ System Labelled Polymer‐HRP anti‐mouse (Dako). Peroxidase binding sites were detected by Vector NovaRED Peroxidase (HRP) Substrate Kit (Vector Laboratories). Harris haematoxylin solution (Sigma‐Aldrich) was used to counterstain liver sections. Labelling index (L.I.) was expressed as the number of BrdU‐positive nuclei/field (at x40 magnification). Ten to 50 fields per liver were scored. A segment of the duodenum, an organ with a high rate of cell proliferation, was used as a positive control for BrdU incorporation.

### 2.8 Statistical analysis

All data were expressed as the mean ± SD. Differences between groups were compared using one‐way analysis of variance ANOVA with the use of GraphPad Software Inc., San Diego, CA. A value of *p* < 0.05 was considered as a significant difference between groups.

### 2.9 Cell culture and in vitro experiments

HepG2 (ATCC, Manassas, VA, USA) and Mahlavu (kindly provided by Dr. N. Atabey) human liver cancer cells were cultured in DMEM complete medium with 10% foetal bovine serum (Lonza, Basel, Switzerland) in a 5% CO_2_ atmosphere. 50×10^3^ cells were transfected using Lipofectamine2000 (Thermo Fisher, Waltham, MA, USA) in a 6 wells plate. Transfection reagents plus miRNA/negative control (hsa‐miR‐106b‐5p #MC10067, Negative Control #4464058) at final concentration of 20nM were used following standard protocols. Seventy‐two hours after transfection, total RNA was extracted with Maxwell^®^ RSC miRNA Tissue Kit (Promega, Madison, WI, USA) according to manufacturer protocol. Total RNA was retro‐transcribed starting from 0.25μg RNA/sample using the High Capacity Kit (Thermo Fisher). Gene expression analysis was performed using the specific TaqMan probes (Thermo Fisher) hCCND1 (hs00765553_m1), and hACTIN (hs99999903_m1) as endogenous control. MiRNA expression was evaluated using the specific Taqman miRNA assay kits (Thermo): hsa‐miR‐106b‐5p #000442 and RNU48 #01006 (as endogenous control). PCR runs were performed with ABI Prism 7900HT (Applied Biosystems).

## 3 RESULTS

### 3.1 Hepatocyte proliferation following PH or TCP treatment.

**FIGURE 1:**
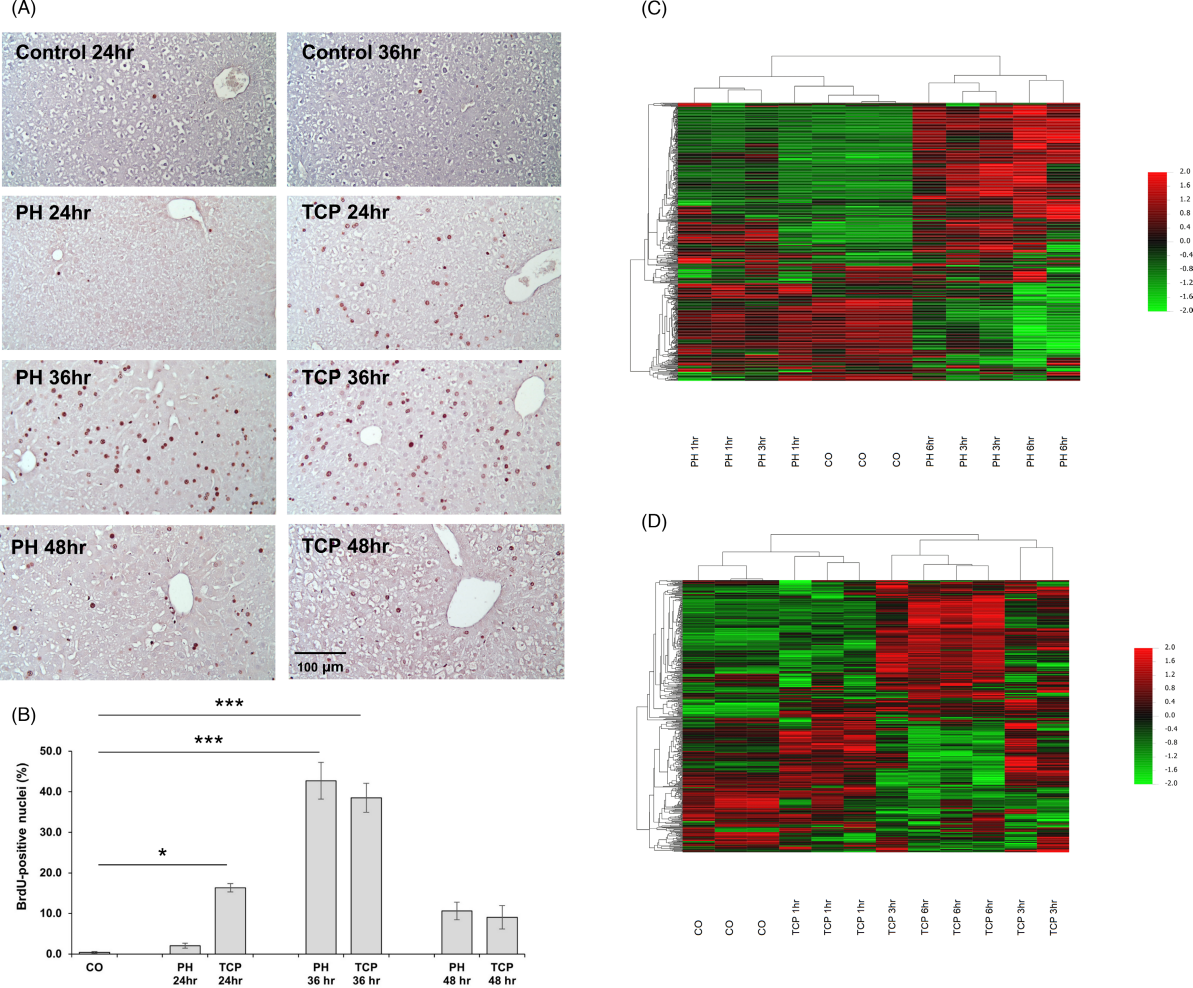
Hepatocyte proliferation and global gene expression profile following PH or TCP treatment. (A) Microphotographs illustrating the presence of BrdU‐positive hepatocyte nuclei (magnification ×20). CD‐1 female mice were subjected to 70% PH or TCP (3 mg/kg body weight) treatment and sacrificed 24, 36 or 48 h thereafter. All animals received BrdU (100 mg/kg) 2 h before the sacrifice; (B) Labelling Index (LI). LI was expressed as number of BrdU‐positive hepatocyte nuclei/100 nuclei. *p < 0.05; ***p < 0.001; (C) Hierarchical clustering of genes at the baseline (CO), 1, 3 and 6 h post‐PH. The red and green colours indicate upregulation and downregulation, respectively. Each row represents the expression of a gene and each column a sample. (D) Heatmap of differentially expressed genes at the baseline (CO), 1, 3 and 6 h post‐TCP. The red and green colours indicate upregulation and downregulation, respectively

According to previous reports,^16^ the measurement of labelling index of hepatocytes from mice subjected to TCP and PH showed that while a high number of hepatocytes was in an active S phase as early as 24 h after TCP treatment, almost no BrdU‐positive cells could be observed at the same time point after PH (Figure 1A, B). However, the peak of DNA synthesis was observed in both groups 36 h after treatment (Figure 1A, B) with a trend towards a return to quiescence at 48 h. Since these data suggest that different molecular events are responsible for the accelerated entry of hepatocytes into S phase observed after TCP treatment, we investigated the possible involvement of miRs in the priming phase of liver cells. To this aim, we analysed the expression profiles of mouse hepatic mRNA and miR at 1, 3 and 6 h after PH or TCP. Identification of miR and mRNA expression abundance was evaluated by NGS (miR) and Illumina microarray (mRNA) in the same samples.

### 3.2 Global gene expression profiles in regenerating livers after PH and TCP.

Global transcriptome changes at 1, 3 and 6 h revealed a total of 810 and 527 genes differentially expressed (DE) in the PH and TCP groups, respectively (Table S1 and S2). Hierarchical clustering analysis of the PH differentially expressed genes stratified them into two major clusters: (1) control liver and PH 1 h and (2) PH 3 and 6 h (Figure 1C). Similarly, it also stratified differentially expressed genes after TCP into two major clusters: (1) control liver and TCP 1 h and (2) TCP 3 and 6 h (Figure 1D).

**FIGURE 2:**
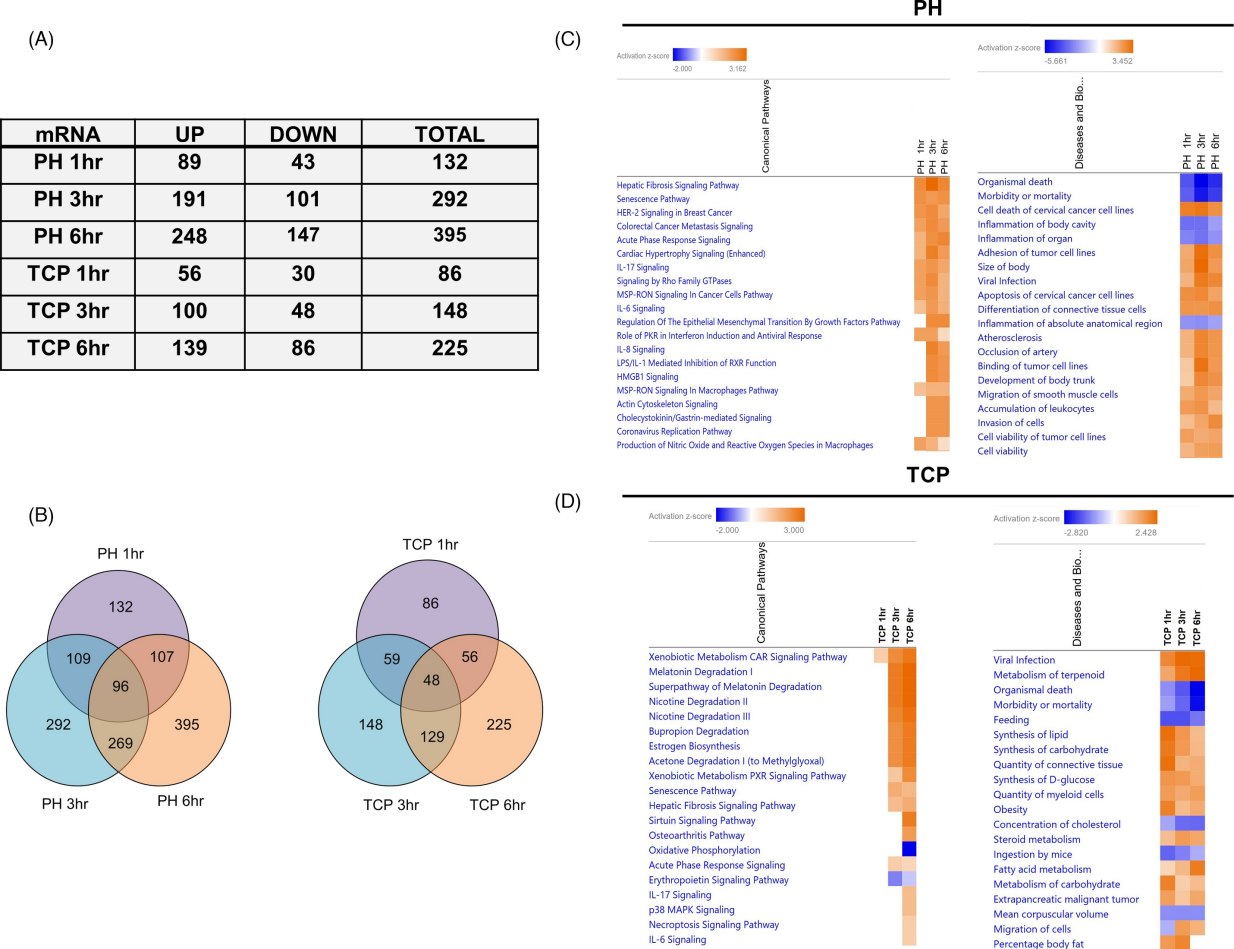
Ingenuity Pathway Analysis of deregulated genes after PH or TCP treatment. (A) Table showing the number of genes up‐ or downregulated at 1, 3 and 6 h post‐PH and TCP treatment; (B) Venn diagrams showing the number of deregulated and overlapping genes at 1, 3 and 6 h post‐PH or TCP treatment; (C) Heatmap for top 20 canonical pathways and diseases and biological function at 1, 3 and 6 h post‐PH. Colour is determined by Z‐score; the Z‐score >2 and <−2 is considered significant. Blue colour indicates suppressed disease /biological function or canonical pathways; orange indicates activated disease/biological function or canonical pathways; (D) Heatmap for top 20 canonical pathways and diseases and biological functions at 1, 3 and 6 h post‐TCP. Colour is determined by Z‐score; the Z‐score >2 and <−2 are considered significant. Blue colour indicates suppressed disease /biological function or canonical pathways; orange indicates activated disease/biological function or canonical pathways

As shown in Figure 2A TCP induced deregulation of a lower number of genes (Figure 2A). The highest number of genes was found to be deregulated at 6 h after PH (395 genes), while a much lower number of genes was deregulated after TCP treatment—148 and 225 genes—at 3 and 6‐h, respectively. Ninety‐six genes resulted commonly altered at all the time points after PH, while 48 were commonly altered at all the time points upon TCP (Figure 2B). Our analysis also showed that 20 genes were commonly deregulated upon both treatments (11 were upregulated, 8 downregulated and 1 exhibited an inconsistent pattern of expression; Figure S1).

Ingenuity Pathway Analysis (IPA) of DE genes 1, 3 and 6 h after PH versus control liver revealed their involvement in pathways related to Hepatic Fibrosis Signaling Pathway and Senescence Pathway (Figure 2C). The functional investigation also underlined common modifications between 1, 3 and 6 h post‐PH (Figure 2C), but none of them was directly related to cell cycle/cell proliferation.

IPA of the genes differentially expressed at 1, 3 and 6 h after TCP vs. control livers displayed completely different pathways and included Xenobiotic Metabolism CAR, Superpathway of Melatonin Degradation as well as Nicotine Degradation (Figure 2D). Functional investigation mainly underlined metabolic pathway modifications such as Synthesis of lipid, Feeding and Synthesis of Carbohydrate (Figure 2D).

**FIGURE 3:**
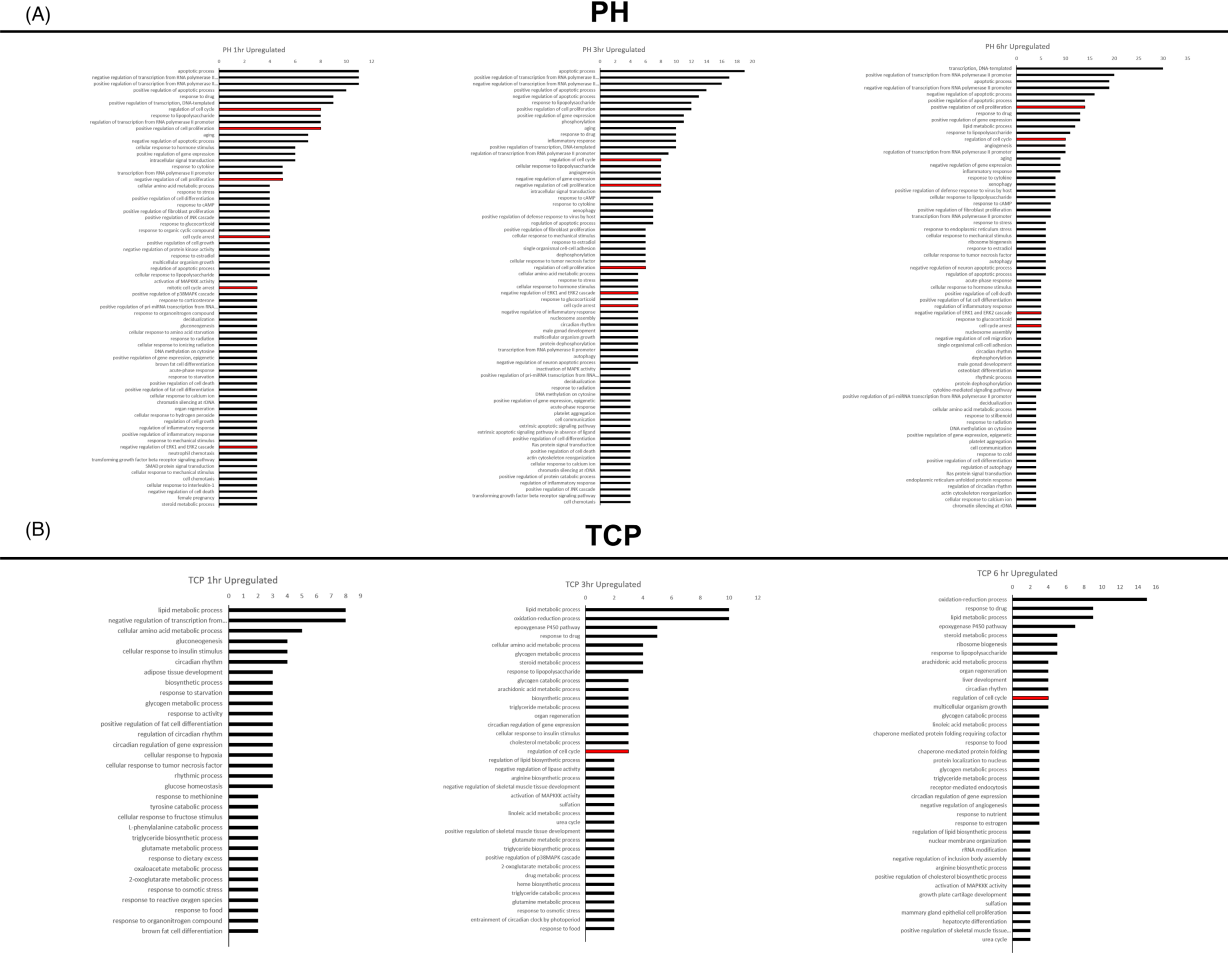
DAVID functional analysis using Gene Ontology annotation. (A) Gene Ontology enrichment analysis of biological processes for upregulated genes at 1, 3 and 6 h post‐PH; (B) Gene Ontology enrichment analysis of biological processes for upregulated genes at 1, 3 and 6 h post‐TCP treatment. Red colour indicates pathways positively/negatively related to regulation of cell proliferation

We also performed DAVID functional analysis using Gene Ontology annotation. As shown in Figure 3A, among the genes upregulated after PH, many were related to apoptosis, cell cycle regulation and cell cycle arrest. Interestingly, genes classified as negative regulators of cell proliferation (*Sox9*, *Cdkn1a*, *Jun*, *Trp53inp1*, *Agt*, *Gja1*, *Tgif1*) or of ERK1/ERK2 cascade (*Ats3*, *Dusp1*, *Dusp6*, *Timp3*, *Ptpn1*) were upregulated following surgery (Figure S2). On the other hand, genes related to cellular metabolism were among the most deregulated after TCP (Figure 3B). Interestingly, only genes positively related to the cell cycle (*Gadd45b*, *Sgk1*, and *Ccnd1*) were observed following xenobiotic treatment (Figure S2). No evidence of increased expression of negative regulators of cell proliferation, cell cycle arrest or of ERK1/ERK2 cascade was observed after TCP (Figure 3B).

The most downregulated pathways in both experimental conditions are listed in Figure S3. While most of them involved metabolic pathways, none was directly related to cell cycle/cell proliferation.

### 3.3 Transcription Factors‐Dependent Pathways

**FIGURE 4:**
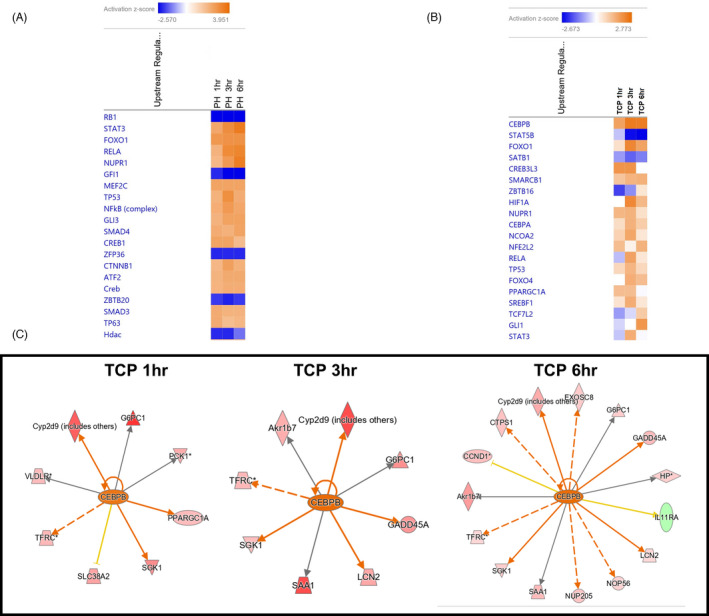
Transcription factor analysis following PH or TCP treatment. (A, B) Heatmaps for top 20 upstream regulators at 1, 3 and 6 h post‐PH (A) or TCP (B). Colour is determined by Z‐score; the Z‐score >2 and <−2 is considered significant. Blue colour indicates suppressed disease/biological function or canonical pathways; orange indicates activated disease/biological function or canonical pathways; (C) Gene interaction networks regulated by the transcription factor CEBPβ in mice 1, 3 and 6 h after treatment with TCP

Next, we analysed transcription factor (TF)‐dependent pathways differentially activated in the livers of PH and TCP mice. By examining the top 20 TFs in each group, we found striking differences between the two proliferative stimuli. Indeed, while RB1 was the most significantly downregulated TF in the PH livers at all the analysed time points it was not listed among the first 20 TFs after TCP (Figure 4A, B). In addition, while STAT3 was among the most significantly upregulated TF after PH (Figure 4A), it was profoundly downregulated 1 h after TCP treatment (Figure 4B). Furthermore, C/EBPβ, a TF that initiates a cascade of gene expression responsible for proliferation,^24^ was strongly upregulated by TCP at all the examined time points, whereas it was not modified within the first 6 h after surgery. As shown in Figure 4C, several CEBPβ‐controlled genes progressively increased in the liver of mice treated with TCP 1, 3 and 6 h after treatment.

Five TFs (STAT3, RELA, NUPR1, FOXO1, TP53) were deregulated in both PH and TCP‐treated mice (compare Figure 4A, B). STAT3 and RELA were strongly upregulated following PH at all the analysed time points, while they were profoundly downregulated 1 h following TCP treatment. These results confirm previous observations showing that no change in the activation of transcription factors implicated in liver regeneration such as, NF‐kB, AP‐1 and STAT3 has been observed in nuclear receptor‐mediated hepatocyte proliferation.^13^ As to FOXO1—strongly upregulated after PH and unchanged or only slightly upregulated post‐TCP—it is interesting to note that forced expression or conditional activation of FOXO factors led to reduced Cyclin D1 expression.^25^, ^26^

### 3.4 miR expression in PH or TCP‐treated mice

**FIGURE 5:**
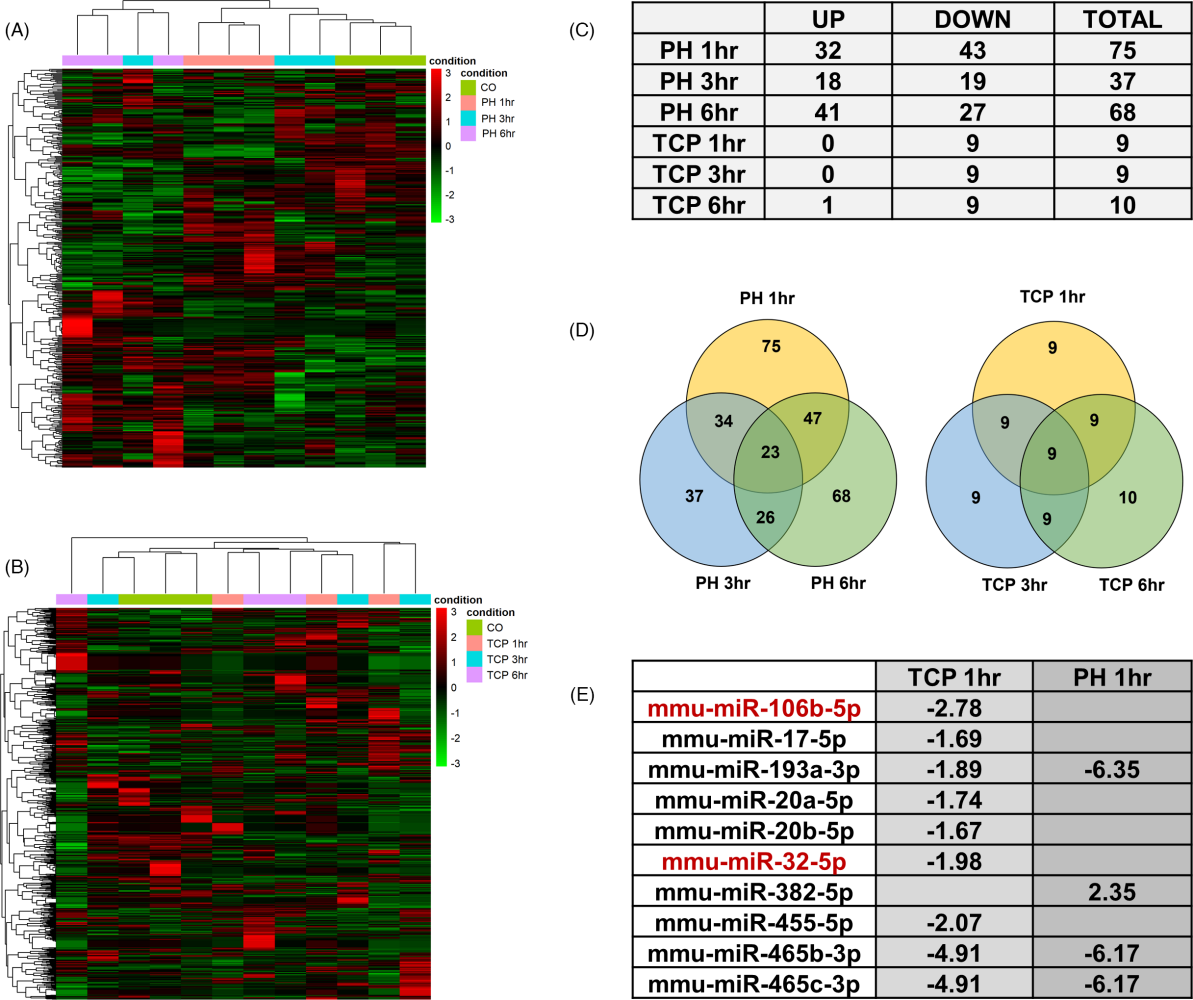
TCP and PH modify the global miR expression profile. (A) Heatmap of differentially expressed miRs at 1, 3 and 6‐h post‐PH. Red and green colours indicate miR upregulation and downregulation, respectively; (B) Heatmap of differentially expressed miRNAs at 1, 3 and 6 h post‐TCP. Red and green colours indicate upregulation and downregulation, respectively; (C) Table showing the number of miRs up or downregulated at 1, 3 and 6 h post‐PH or TCP treatment; (D) Venn diagrams showing the number of deregulated and overlapping miRs at 1, 3 and 6 h post‐PH or TCP treatment; (E) Table indicating miRs deregulated by TCP at 1 h, in common with PH. In red are indicated the miR deregulated exclusively in the TCP‐treated mice

To investigate miRs differentially expressed in the liver following the two proliferative stimuli, we applied time‐course analysis using the R/Bioconductor package “DESeq2”. Hierarchical clustering analysis in the PH group stratified the three time points into 2 major clusters: 1) Controls (CO), PH 1 and 3 h and 2) PH 6 h. Hierarchical clustering analysis on TCP samples did not display a clear separation of the different time points (Figure 5A, B).

A striking difference in the number of differentially expressed miRs was found between the two proliferative conditions (Figure S4 and S5). Indeed, while the expression of 102 miRs was significantly modified after PH, only 10 miRs were found differentially expressed in the TCP group. As to PH, the Venn diagram showed that the highest number of miRs was deregulated at 1 h (75 miRs) and 6 h (68 miRNAs) post‐surgery, whereas 23 miRs resulted commonly altered at all the time points (Figure 5C, D). Notably, the three most upregulated miRs 1 h after PH, miR‐124‐3p, miR‐9‐3p and miR‐9‐5p (Figure S4) act as inhibitors of proliferation in several cell types.^27^, ^28^

Similar to what was observed with mRNA expression, a much lower number of deregulated miRs was found after TCP treatment at all the analysed time points, with only 9 miRs being commonly altered at all the time points. Differently from PH, TCP caused downregulation of all the miRs at each time point, with the only exception of miR‐382‐5p that was upregulated 6 h after administration of the drug (Figure 5E, Figure S5). Interestingly, out of the 9 miRs deregulated 1 h after TCP, only 3 were altered in PH livers (Figure 5E). Notably, while most miRs were altered in both experimental groups (Figure S4 and S5), only 2 miRs—miR‐106b‐5p and miR‐32‐5p—were exclusively deregulated in TCP‐treated mice (Figure 5E).

### 3.5 miR‐mRNA interactions

To identify a possible link between differentially expressed miRs and genes, among the identified dysregulated genes, we selected multiMiR validated/predicted miR targets. In both experimental groups, several genes associated to the cell cycle and differentially expressed in both experimental groups were indeed targets of deregulated miRs. Among the most downregulated miRs post‐PH, we found 4 miRs (miR‐106a‐5p, miR 340‐5p, miR‐19b‐3p and miRNA‐455‐5p), targeting Socs3, a known tumour suppressor and an inhibitor of cell cycle. Indeed, Socs3 expression was already significantly upregulated 1 h after PH and remained elevated until 6 h post‐surgery (Table S3).

**FIGURE 6:**
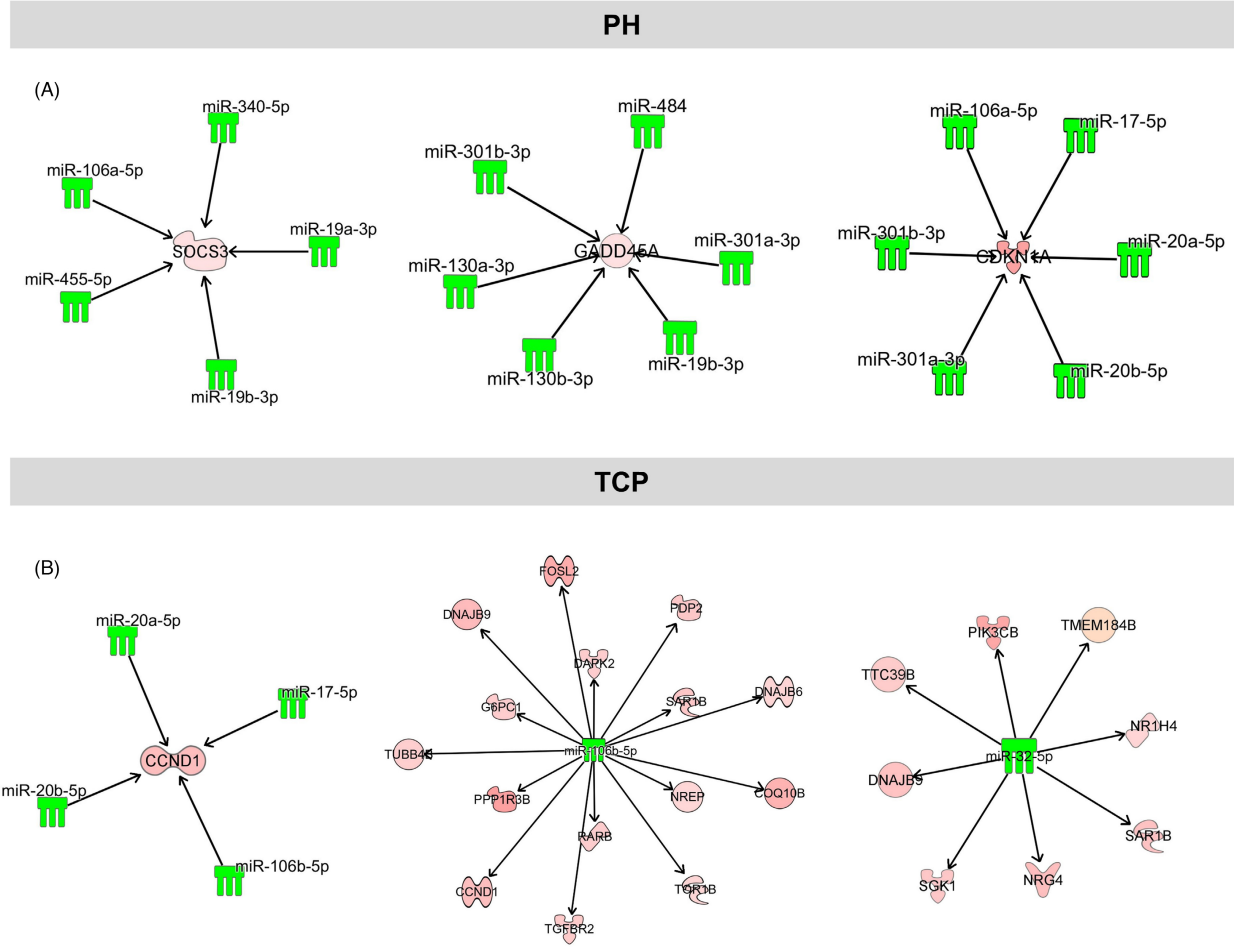
mRNA‐miR interaction networks of cell cycle‐related genes in mice subjected to PH or treated with TCP. (A) mRNA‐miR networks of *Socs3*, *Gadd45a* and *Cdkn1a* in mice subjected to PH showing that downregulation of several miRNAs is associated with the upregulation of their target genes; (B) mRNA‐miR networks of *Ccnd1*‐*miR*‐*106b*‐*5p and Sgk1*‐*Pik3cb*‐miR‐32‐5p in TCP‐treated mice. The panel shows miRs predicted/validated to target *Ccnd1* and downregulated after TCP (left side), and all the predicted target genes of miR‐*106b*‐*5p* and *miR*‐*32*‐*5p –*the only two miRs differentially expressed only in TCP mouse liver (middle and right side)

After PH, we also found upregulated both genes considered to be positively correlated to induction of proliferation (ie *c*‐*fos* and *Ccnl1*), as well as negative regulators of cell cycle (*Gadd45a and Cdkn1a*). In particular, Gadd45a was upregulated at all the time points and its upregulation was paralleled by downregulation of miR‐301b‐3p, miR‐484 and miR‐19b‐3p (validated) and miR‐130a‐3p, miR‐130b‐3p and miR‐301‐3p (predicted) to target it, as early as 1‐h post‐surgery. Moreover, downregulation of miR‐301 was associated with the upregulation of its target gene *Cdkn1a* (Figure 6A and Table S3).

Nfkbiz—another negative regulator of the cell cycle^12^, ^29^ was upregulated 3 and 6 h after PH. Interestingly, in rat liver Nfkbiz is a target of miR‐376b.^30^ In our study, however, the role of miR‐376b is unclear as it was upregulated at 1 and 3 h after PH and downregulated at 6 h (Table S3). Whether mouse Nfkbiz is a target of miRs other than miR‐306b will require further studies.

In the TCP experimental group, no cell cycle negative regulator—other than Gadd45a—was significantly modified compared to control liver. Remarkably, *Ccnd1*, the gene encoding for cyclin D1, responsible for the G0‐G1 transition, was upregulated 6 h after TCP treatment. Such upregulation was paralleled by downregulation of cyclin D1 targeting miRs (miR‐20a‐5p, miR‐20b‐5p and miR‐17‐5p) as early as 1 h after TCP (Figure 6B). Notably, after PH, downregulation of the same miRs was observed only at 6 h (Figure S4). Only 2 miRs resulted exclusively deregulated in the liver of TCP‐treated mice: miR‐106b‐5p, predicted to target *Ccnd1*, which was downregulated at all the time points, and miR‐32‐5p targeting other positive regulators of the cell cycle, namely *Sgk1* and *Pik3c*b^31^, ^32^ (Figure 6B; Table S3). To validate the NGS results, we performed qRT‐PCR analysis on a selected set of genes and miRNAs. As shown in Figure S6A, the expression of all the investigated genes (*Ccnd1*, *Cdkn1a*, *Cype2b10*, *Socs3*, *Gadd45b* and *Gadd45a*) was deregulated similar to what observed by NGS.

QRT‐PCR analysis was also performed to validate the changes in the levels of miR‐106b‐5p predicted to target *Ccnd1* and whose expression was downregulated after TCP (Figure 5E) and of miR‐301b and miR‐455—predicted to target *Cdkn1a* and *Socs3*, respectively and found upregulated after PH (Figure S4). As shown in Figure S6B, the expression of these miRs was deregulated in accord to NGS results.

Next, to further validate the effect of miR‐106b‐5p on *Ccnd1* we transduced two human liver carcinoma cell lines (Mahlavu and HepG2) with this miRNA and measured the mRNA levels of its target gene. As shown in Figure S7, expression of miR‐106b‐5p led to a significant downregulation of *Ccnd1*.

## 4 DISCUSSION

In the present study, we unveiled the miR‐mRNA networks involved in the priming phase of hepatocyte proliferation elicited by stimuli of different nature: PH, which stimulates liver regeneration, and TCP, which induces direct hyperplasia. To this aim, we evaluated the expression profiles of mRNAs and miRs at 1, 3 and 6 h after surgery or treatment with TCP.

Several studies reported early response of the mouse liver transcriptome following treatment with TCP^17^, ^18^ or after PH,^12^ but none of the researches conducted so far correlated gene expression changes with miR profile in the same samples in both the experimental conditions. Indeed, previous studies in the literature reported deregulation of some miRs in the priming, proliferative and termination phases upon PH, but the results were often contradictory due to differences in strain, age and timing of observation.^4^, ^5^, ^6^, ^33^, ^34^, ^35^, ^36^ As to TCP, few studies have investigated the role of miRs in hepatocyte proliferation, but none of them analysed early time points post‐treatment.^37^, ^38^ Thus, the key strength of our study is that these two experimental protocols (liver regeneration post‐PH and direct hyperplasia following TCP treatment) were performed in parallel on mice of the same strain, gender and age and at the same time. Moreover, we analysed both miRs and mRNAs in the same samples to generate a network of interactions, possibly explaining the transcriptomic modifications driving the two different proliferative modalities.

A much higher number of genes and miRNAs was found deregulated after PH when compared to TCP. The difference was particularly striking for miRNAs (102 post‐PH vs. 10 after TCP). In this context, it is important to stress that liver regeneration after 2/3 PH is part of a complex interplay of distinct sets of rapidly evolving changes, such as those caused by the metabolic and circulatory perturbations consequent to the removal of 2/3 of the liver and mitogenic changes. This makes difficult to discriminate genes and miRNAs directly related to the entry into the cell cycle from those responsible for the adjustments of essential hepatic functions. Such a massive metabolic rearrangement is clearly not required by the liver following a single treatment with TCP, as the liver does not have to compensate for a reduced size. It is possible that while the vast majority of miRNAs play a relevant role in the metabolic rearrangement of the remnant liver post‐PH and in the quite stressful condition associated to the surgical procedure, this is not the case after TCPOBOP, where only few miRNAs—some of which targeting cell cycle genes—are downregulated.

The most important findings of the present work are: 1) many genes functioning as negative regulators of the cell cycle were upregulated after PH, but not after TCP; 2) miRs negatively controlling cell cycle genes were downregulated only after surgery; 3) miRs predicted to target *Ccnd1*, such as miR‐106b‐5p, were significantly downregulated only in TCP‐treated mice.

Previous studies demonstrated that, in mice, the entry of hepatocytes into the S phase of the cell cycle is robustly anticipated in TCP‐treated animals compared to PH.^16^ Indeed, an active DNA synthesis takes place 24 h after TCP, a time when virtually no dividing hepatocytes can be seen in livers undergone PH. In this context, it is interesting to note that Cyclin D1 induction occurs early after treatment with TCP, but not after PH.^16^ For this reason, the identification of *Ccnd1* targeting miRs already downregulated 2 h after TCP may explain the increase of cyclin D1 and the consequent faster entry into the cell cycle. Importantly, miR‐106b‐5p, predicted to target *Ccnd1*, was indeed exclusively downregulated after TCP treatment, at all the time points analysed. A second miR, miR‐32‐5p, was similarly downregulated only after TCP; this miR targets other positive regulators of the cell cycle, such as Sgk1 and Pik3cb, whose expression was increased in the same samples following TCP treatment. In agreement with Yin et al.^12^ following surgery we observed downregulation of miRs targeting Socs3, a known tumour suppressor and a cell cycle inhibitor. In addition, we also found increased levels of the cell cycle inhibitor *Cdkn1a*, preceded by downregulation of miR‐301, targeting *Cdkn1a*. Furthermore, we observed a strong upregulation of the oncosuppressor miR‐34 family. Of note, the increased expression of miR‐34 observed during the termination phase of liver regeneration has been reported as a potential ‘stop’ signal.^39^ This finding together with the report of Sun^40^ showing that miR‐34a targets the 3’ untranslated mRNA region of *Ccnd1*, supports the concept that an active control on mitogenic signals operates after PH, thus leading to a delay in the entry into the cell cycle. Conversely, no evidence of miR‐34 deregulation was observed at any time point in TCP‐treated livers (Figure S4).

Another interesting observation that could justify the different kinetics of S phase entry of the two proliferative stimuli is the finding that while after PH pathways related to inflammatory response were among the most deregulated, pathways involving upregulation of metabolic changes, especially lipid metabolism—required for sustaining cell proliferation—were the most modified after TCP.

In conclusion, the analysis of the microRNAs‐mRNA networks performed in the present study unveils on the one hand that different miRs are implicated in the early phase of hepatocyte proliferation induced by mitogenic stimuli of different nature, and on the other hand that miRs, such as miR‐106b‐5p are critical in regulating the levels of the main cyclin implicated in the G1‐S transition of the cell cycle.

A limitation of this study relies on the impossibility to functionally validate some of the present findings since primary hepatocytes in vitro do not express CAR and do not proliferate after TCP.^41^ Nevertheless, the present work, performed in a strictly controlled experimental condition, contains a number of novel findings that can be helpful for a better comprehension of the molecular mechanisms responsible for deciphering proliferative signals in totally diverse conditions, such as those where hepatocyte replication is needed to replace cell loss (ie PH or chemically induced necrosis) or those where proliferation occurs in the intact liver (TCP as well as other hepatomitogens, such as T3 and PPAR ligands).

## CONFLICT OF INTEREST

All authors declare that they have no conflict of interest.

## AUTHOR CONTRIBUTION

Rajesh Pal and Marta Anna Kowalik conducted research and drafted the manuscript. Marina Serra provided assistance in the process of revised drafting manuscript and figure and tables construction. Cristina Migliore performed in vitro experiments. Amedeo Columbano, Andrea Perra and Silvia Giordano contributed to conceptual framework, supervised the study and revised the manuscript.

## Supporting information

Figure S1

Figure S2

Figure S3

Figure S4

Figure S5

Figure S6

Figure S7

Table S1

Table S2

Table S3

Supporting Information

## Data Availability

The data that support the findings of this study are available from the corresponding author upon reasonable request. Raw data can be accessed in following IDs: GEO accession No (mRNA): GSE185316. SRA ID (miRNA): PRJNA769011.
