# Supplementary figures and images for: Diverse MicroRNAs‐mRNA networks regulate the priming phase of mouse liver regeneration and of direct hyperplasia
Source: Cell Prolif. 2022 Feb 17;55(4):e13199. doi: 10.1111/cpr.13199 (PMC9055901; doi:10.1111/cpr.13199)

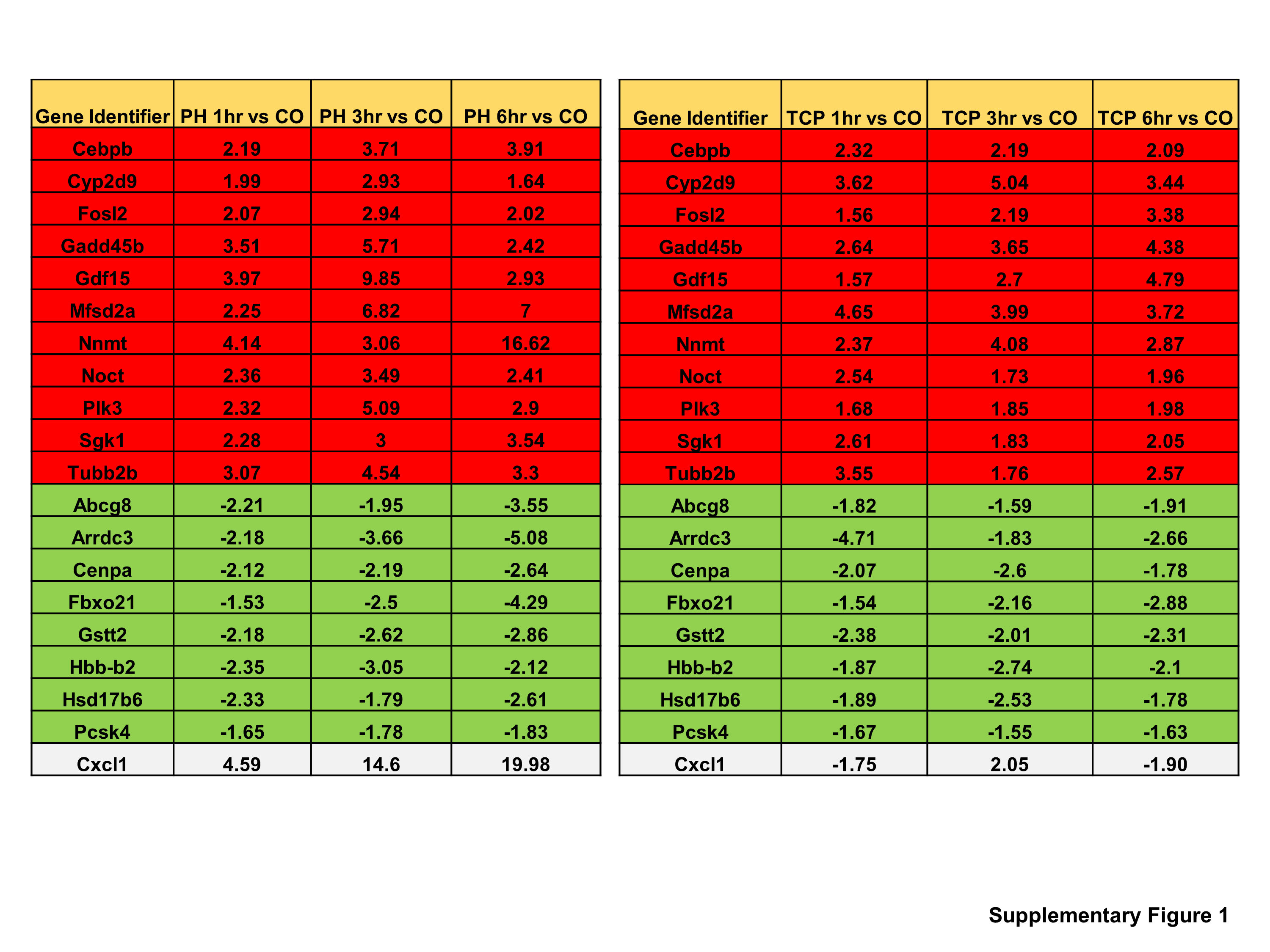

Supplement: Supplementary file 1 — Figure S1 [file CPR-55-e13199-s005.tif]

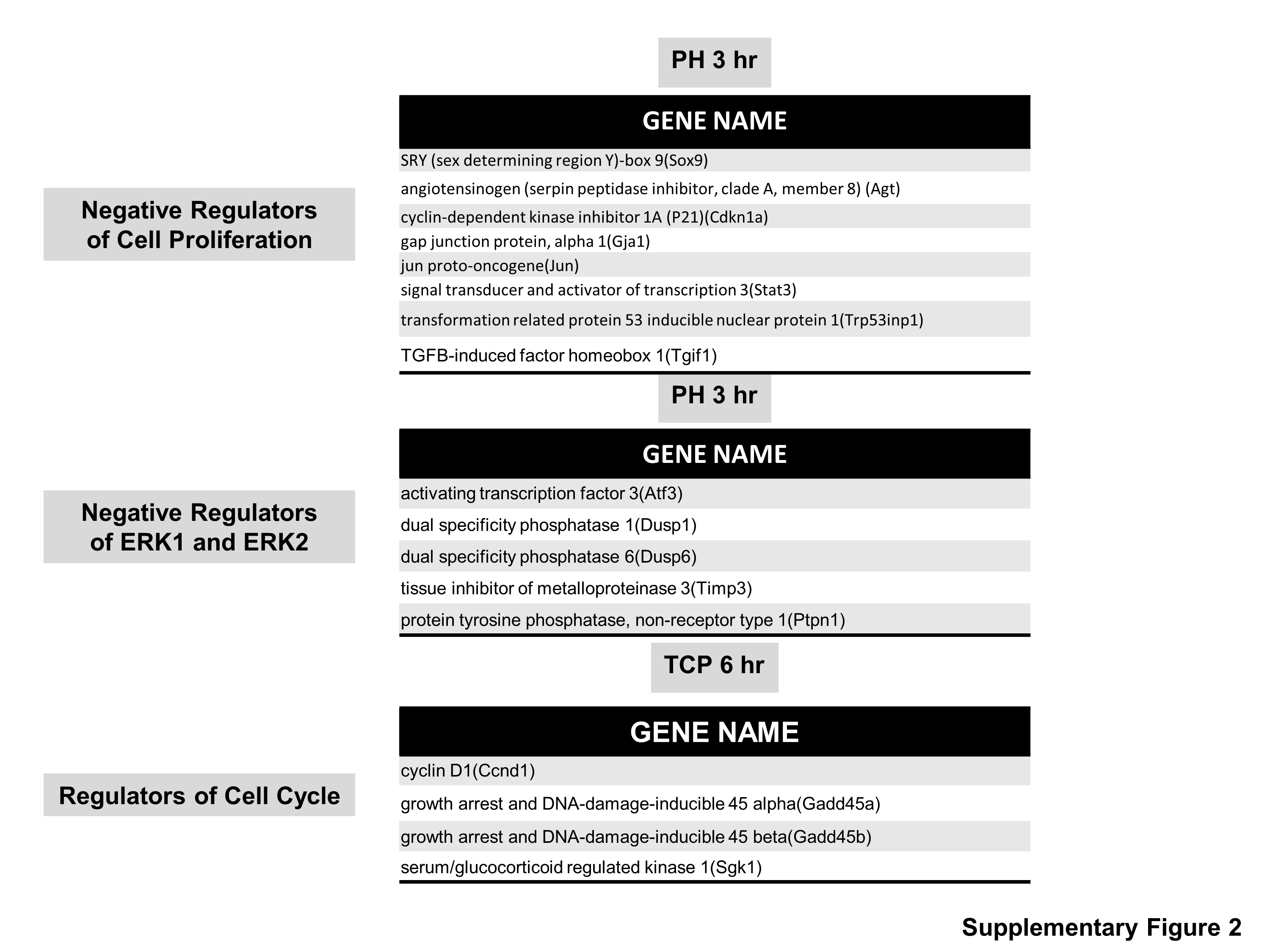

Supplement: Supplementary file 2 — Figure S2 [file CPR-55-e13199-s008.tif]

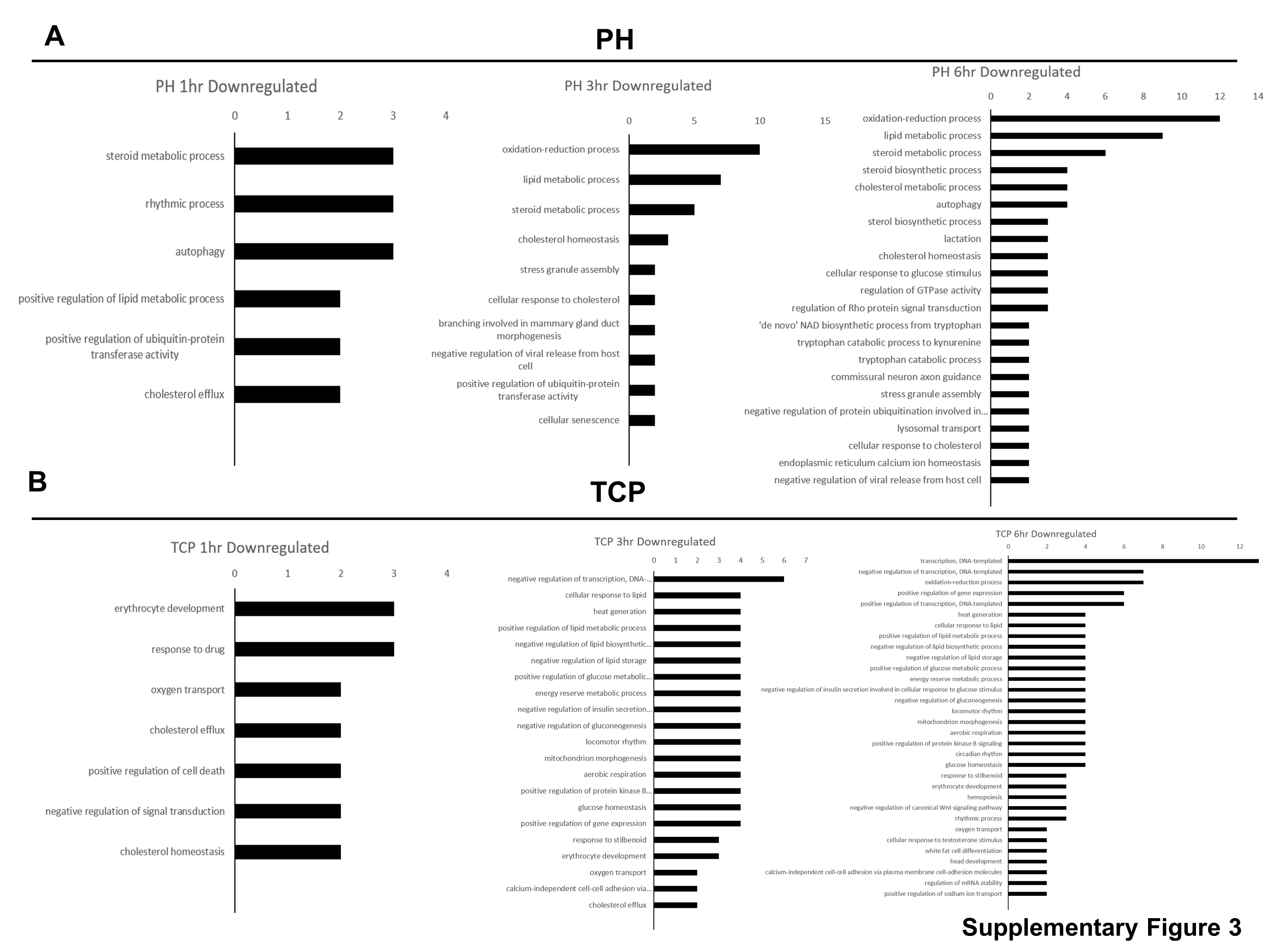

Supplement: Supplementary file 3 — Figure S3 [file CPR-55-e13199-s010.tif]

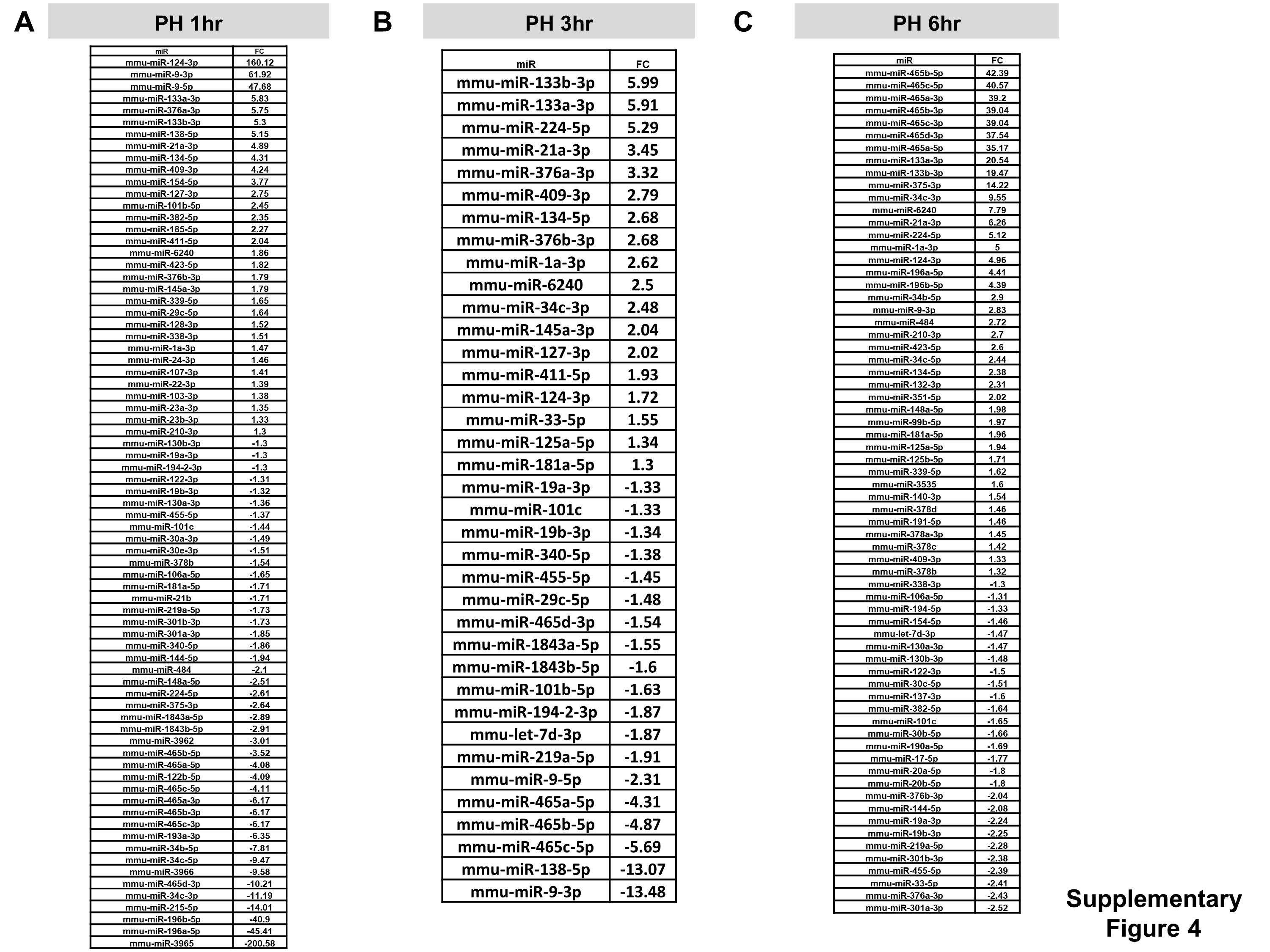

Supplement: Supplementary file 4 — Figure S4 [file CPR-55-e13199-s003.tif]

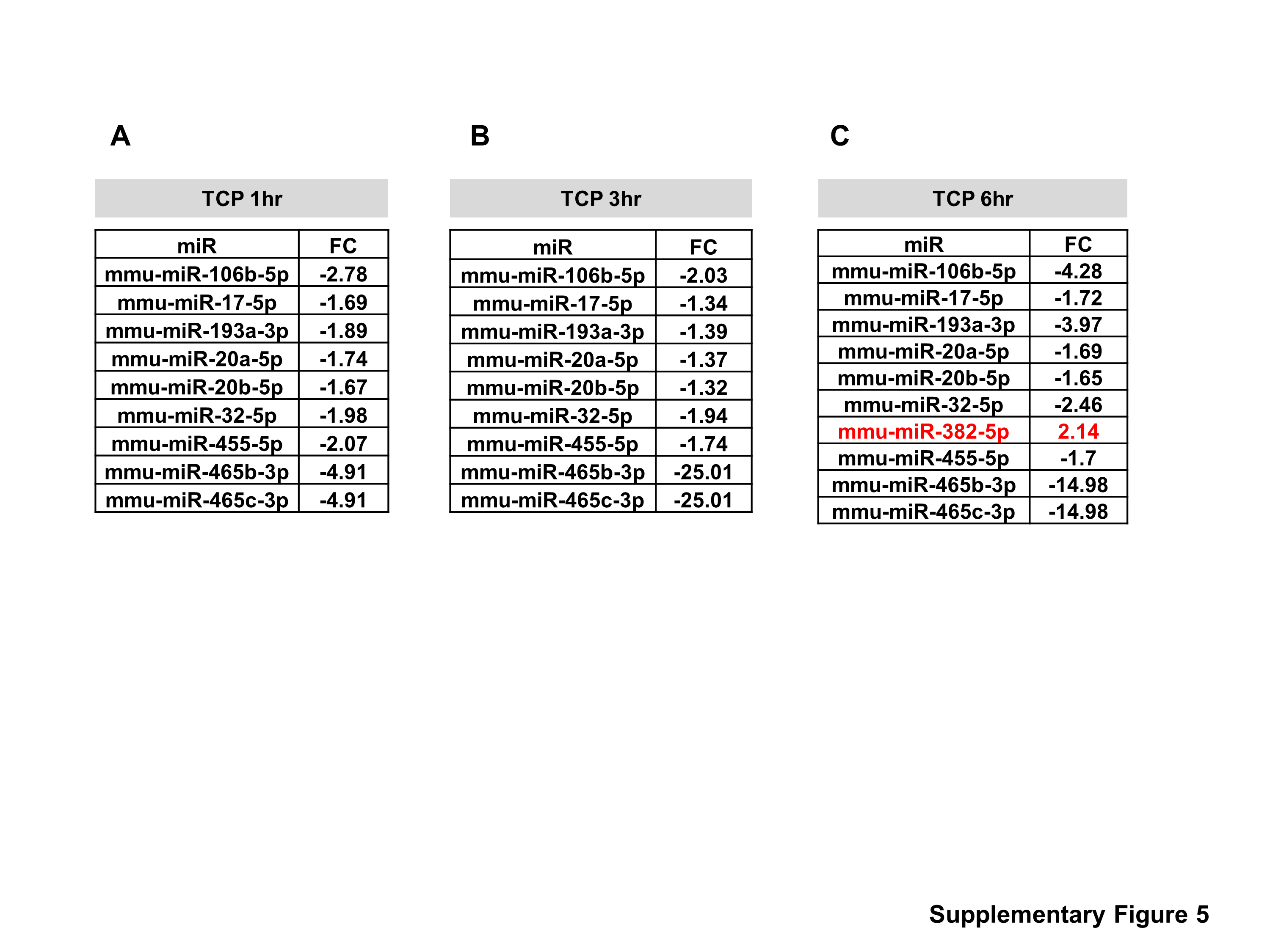

Supplement: Supplementary file 5 — Figure S5 [file CPR-55-e13199-s002.tif]

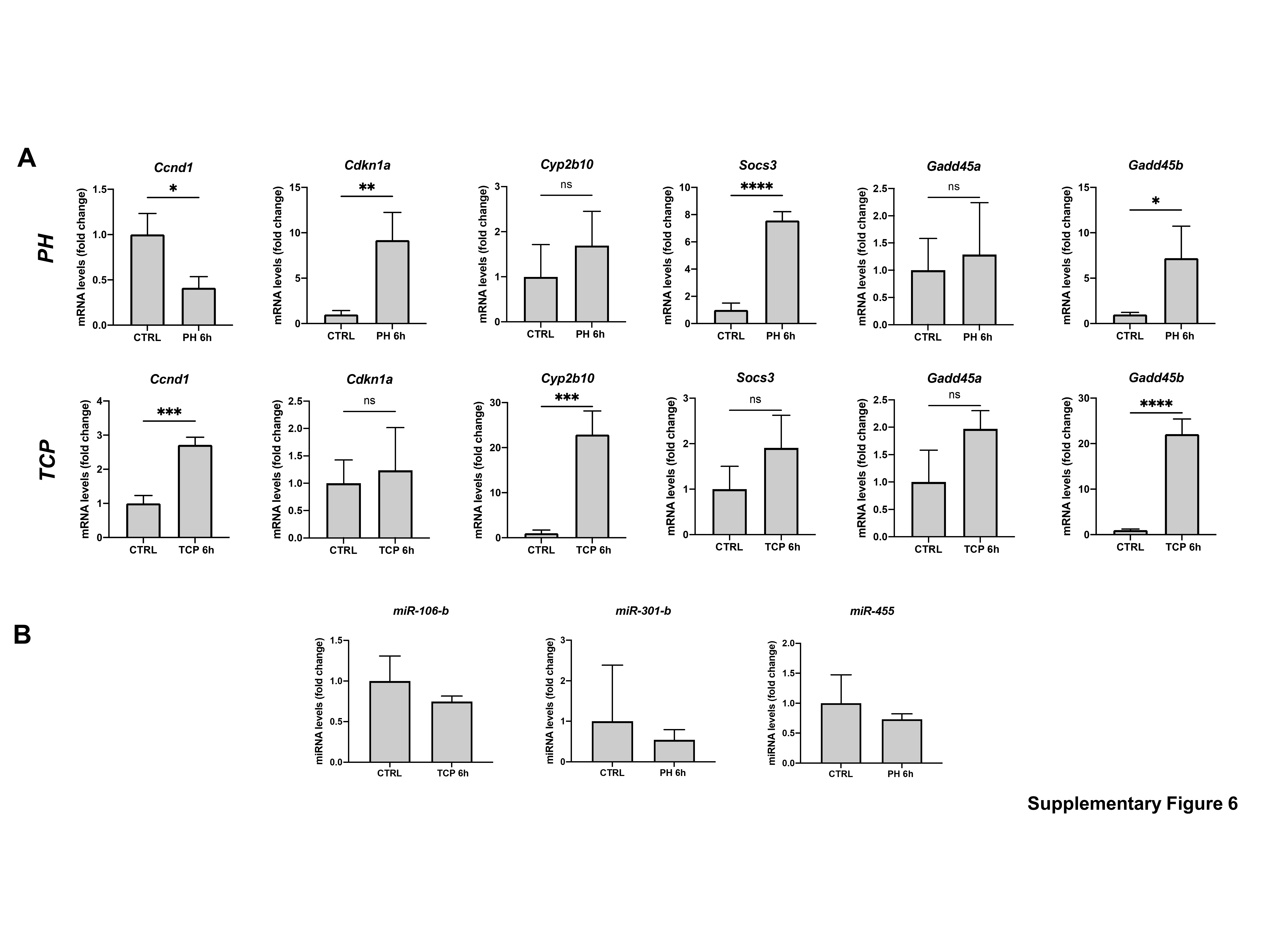

Supplement: Supplementary file 6 — Figure S6 [file CPR-55-e13199-s011.tif]
