# Supplementary material for: Diverse MicroRNAs‐mRNA networks regulate the priming phase of mouse liver regeneration and of direct hyperplasia
Source: Cell Prolif. 2022 Feb 17;55(4):e13199. doi: 10.1111/cpr.13199 (PMC9055901; doi:10.1111/cpr.13199)
Supplement: Supplementary file 11 — Supporting Information [file CPR-55-e13199-s001.pdf]

Supplementary Figure 1. Global gene expression changes following PH or TCP treatment.

Up- and down-regulated genes following PH and TCP treatment at 1, 3 and 6 hours. Red and green colors indicate up-regulation and down-regulation, respectively, in both experimental groups. The grey color indicates genes that do not show a similar expression pattern in both groups (FC ±1.5, P value ≤0.05).

Supplementary Figure 2. PH and TCP treatment affect the pathways related to cell proliferation.

Identification of genes categorized as Negative Regulators of Cell Proliferation and Negative Regulators of ERK1 and ERK2 deregulated 3 hours post PH. Identification of genes belonging to the category of Regulators of Cell Cycle deregulated 6 hours post TCP. (According to DAVID Functional Annotation)

Supplementary Figure 3. DAVID functional analysis using Gene Ontology annotation.

A,B) Gene Ontology enrichment analysis of biological processes for down-regulated genes at 1, 3 and 6 hours post PH (**A**) or TCP **(B)**.

Supplementary Figure 4. List of the differentially expressed miRs following PH.

A list of the up and down-regulated miR at 1, 3 and 6 hours post-PH (FC ±1.3, P value ≤0.05).

Supplementary Figure 5. List of the differentially expressed miRs following TCP.

A list of the up and down-regulated miRs at 1, 3 and 6 hours post TCP (FC ±1.3, P value ≤0.05). The only up-regulated miR (miR-382-5p) is indicated in red.

Supplementary Figure 6. QRT-PCR validation of selected genes and miRs.

A) Differentially expressed genes after PH or TCP Gene expression is reported as fold change relative to livers from untreated mice. Results are expressed as means ± SD. *$P<0.05$; **$P<0.01$, ***$P<0.001$; **B)** QRT-PCR analysis of miR-106b, miR-301b and miR-455 in mouse liver following PH or TCP. Gene expression is reported as fold change relative to livers from untreated mice**.**

Supplementary Figure 7. Effect of miR-106 on Ccnd1 expression in human cell lines. **A)** QRT-PCR analysis of miR-106 levels in HepG2 and Malhavu hepatoma cells following miRNA transfection. MiRNA expression is reported as fold change relative to untreated cells. ***P<0.001; **B)** Effect of transfection of miR-106 on *Ccnd1* expression. Gene expression is reported as fold change relative to untreated cells. Results are expressed as means ± SD. *P<0.05.

Supplementary Table 1. List of differentially expressed mRNA at 1, 3 and 6 hours post-PH (FC ±1.5, P value ≤0.05).

Supplementary Table 2. List of differentially expressed mRNA at 1, 3 and 6 hours post-TCP (FC ±1.5, P value ≤0.05).

Supplementary Table 3. mRNA-miR interaction networks at 1-, 3- and 6-hours post-PH or TCP treatment. Validated and predicted interactions are indicated.
